# Assessment of Spending for Patients Initiating Dialysis Care

**DOI:** 10.1001/jamanetworkopen.2022.39131

**Published:** 2022-10-28

**Authors:** Riley J. League, Paul Eliason, Ryan C. McDevitt, James W. Roberts, Heather Wong

**Affiliations:** 1 Department of Economics, Duke University, Durham, North Carolina; 2 Department of Economics, Brigham Young University, Provo, Utah; 3 Fuqua School of Business, Duke University, Durham, North Carolina; 4 National Bureau of Economic Research, Cambridge, Massachusetts

## Abstract

**Importance:**

Despite a widespread belief that private insurers spend large amounts on health care for enrollees receiving dialysis, data limitations over the past decade have precluded a comprehensive analysis of the topic.

**Objective:**

To examine the amount and types of increases in health care spending for privately insured patients associated with initiating dialysis care.

**Design, Setting, and Participants:**

A cohort study covering calendar years 2012 to 2019 included patients with kidney failure who had employer-sponsored insurance for 12 months following dialysis initiation. Data analysis was performed from August 27, 2021, to August 18, 2022. The data cover the entirety of the US and were obtained from the Health Care Cost Institute. The data include all medical claims for enrollees in employer-sponsored health insurance plans offered by multiple major health care insurers within the US. Participants included patients younger than 65 years who were continuously enrolled in these plans in the 12 months before and after their first claim for dialysis care. Patients also had to have nonmissing documented key characteristics, such as sex, race and ethnicity, and health characteristics.

**Exposures:**

A claim for dialysis care.

**Main Outcomes and Measures:**

Out-of-pocket, inpatient, outpatient, physician services, prescription medication, and total health care spending. The hypothesis tested was formulated before data collection.

**Results:**

The sample included 309 800 enrollee-months, which was a balanced panel of 25 months for 12 392 enrollees. At baseline, 7534 patients (61%) were male, 5415 (44%) were aged 55 to 64 years, and patients had been enrolled with their insurer for a mean of 30 months (95% CI, 29.9-30.1 months). In the 12 months before initiating dialysis care, total monthly health care spending was $5025 per patient per month (95% CI, $4945-$5106). Dialysis care initiation was associated with an increase in total monthly spending of $14 685 (95% CI, $14 413-$14 957). This increase occurred across all spending categories (dialysis, nondialysis outpatient, inpatient, physician services, and prescription drugs). Monthly patient out-of-pocket spending increased by $170 (95% CI, $162-$178). These spending increases occurred abruptly, beginning about 2 months before dialysis initiation, and remained increased for the subsequent 12 months.

**Conclusions and Relevance:**

In this cohort study, evidence that private insurers experience significant, sustained increases in spending when patients initiated dialysis was noted. The findings suggest that proposed policies aimed at limiting the amount dialysis facilities charge private insurers and the enrollees has the potential to reduce health care spending in this high-cost population.

## Key Points

**Question:**

Does health care spending on patients with private insurance who have kidney failure change immediately after they initiate dialysis care?

**Findings:**

This cohort study including 12 392 patients noted the initiation of dialysis care for privately insured patients with kidney failure was associated with significant increases in monthly spending from $5025 to $19 654. The differences in spending between patients receiving Medicare compared with private insurance were large: patients insured by Medicare had annual mean spending of $80 509 compared with $238 126 for privately insured patients in their first year of dialysis.

**Meaning:**

The findings of this study suggest that initiating dialysis is associated with large increases in health care spending for patients with kidney failure who are privately insured compared with those receiving Medicare.

## Teaser

This cohort study examines the costs of dialysis for patients with private insurance vs Medicare.

## Introduction

Although previous work has shown that Medicare spends large amounts on patients with kidney failure, comparatively few studies have documented spending patterns by private insurers.^1,2,3,4^ In this study, we analyzed claims data for several thousand privately insured patients receiving dialysis. Building on previous studies, we examined how spending evolves as patients start dialysis, comparing patients receiving dialysis with themselves at varying points in time rather than across different patients who may reflect a different underlying population.

As treatment for a chronic condition, dialysis care is expensive: annual Medicare spending per patient receiving hemodialysis was $93 200 in 2018.^5^ Like many areas of health care, spending by private insurers may be even higher.^6,7^ In 2007, for example, total monthly health care spending was approximately twice as high for privately insured patients just after they started dialysis compared with those insured by Medicare; the same was true of per-person per-year net cost in 2011, a statistic that we updated in this study using contemporary data.^1,2^ Previous research has reported that the median price paid by employer-sponsored plans for a single hemodialysis session is more than 6 times the base rate paid by Medicare for the same service.^8^ This price difference may not be representative of other health care services rendered to privately insured patients receiving dialysis, however, so overall health care spending on these patients remains inconclusive. Furthermore, private insurers may differ from Medicare in their ability to manage nondialysis services, potentially leading to differences in overall spending.

The cost differences between Medicare and private insurance to manage the care of patients receiving dialysis are particularly important because, unlike other conditions, patients diagnosed with kidney failure who require dialysis are automatically eligible for Medicare coverage after a 3-month waiting period, regardless of age.^9^ Furthermore, after a 30-month coordination period during which Medicare serves as a secondary payer for patients with private insurance, Medicare becomes the primary payer. Given the recent Supreme Court decision on the Medicare Secondary Payer Act, the cost differences between these programs remain important for policy makers.^10^

Despite a large and increasing share of patients receiving dialysis enrolled in non-Medicare plans,^11^ few recent studies have examined the cost of care in this population. Although a pair of investigations have assessed spending on hemodialysis care,^2,3^ to our knowledge, the only recent study to examine total health care spending for patients requiring dialysis was conducted by Trish et al,^4^ with the authors reporting that patients with kidney failure enrolled in individual market plans had spending 33 times higher than those without failure. Our study builds on theirs in 3 ways.

First, we used data from employer-sponsored plans, which means our study reflects the most common source of private insurance. Second, we documented the cost of dialysis care by comparing health care spending for patients before and after they start dialysis. Patients with kidney failure may require more medical care than the general population beyond what is needed to replace kidney function. For example, in patients with incident kidney failure, the primary cause was diabetes in 46.7% and high blood pressure in 27.9%; 41.6% of the patients had obesity.^12,13^ Comparing the total health care spending of patients who receive dialysis care with those who do not likely overstates the increase in spending associated with initiating dialysis. We overcome this issue by comparing spending for the same patients before and after their first dialysis session. Third, we examined the differences in spending in greater detail, going beyond a simple comparison of mean spending. We investigated the types of spending that led to the difference and highlight changes across the entire distribution of spending.

## Methods

Our primary data were provided by the Health Care Cost Institute, a nonprofit research institute supported by Aetna, Humana, Kaiser Permanente, and Blue Health Intelligence.^14^ The data include all medical claims for enrollees in employer-sponsored health insurance plans offered by these carriers and cover more than 55 million individuals per year from 2012 to 2019, or approximately one-third of the employer-sponsored insurance market. To account for inflation over our sample period, we denominate all spending in 2019 prices. Data analysis was performed from August 27, 2021, to August 18, 2022.

This study was approved by the institutional review board of Duke University and follows the Strengthening the Reporting of Observational Studies in Epidemiology (STROBE) reporting guideline for cohort studies. More details on the methods, including mathematical expressions of our regression equations and detail on our use of the US Renal Data System data, are available in the eMethods in the Supplement.

To create a sample of patients initiating dialysis care, we identified all outpatient claims indicating dialysis care. Specifically, we identified outpatient claims reporting *Current Procedure Terminology* codes 90935, 90937, 90945, 90947, 90951-90970, 90989, 90999, 99512, or G0257 or revenue codes 0821, 0831, 0841, 0851, or 0881.

We then limited the sample to patients younger than 65 years who were continuously enrolled in plans covered by the facilities included in the 12 months before and after the enrollee’s first claim for dialysis care. We dropped all enrollees for whom we did not observe all patient and plan characteristics reported in Table 1. The data provided to us by the Health Care Cost Institute did not include any information on race and ethnicity. Our results are robust to including patients with missing characteristics. eTable in the Supplement presents the patient characteristics reported in Table 1 without restricting the sample. We noted no meaningful differences between the samples. These restrictions resulted in a sample of 309 800 enrollee-months—a balanced panel of 25 months for 12 392 enrollees.

**Table 1.** Predialysis and Postdialysis Summary Statistics^a^

Variable	Predialysis, mean (SD)	Postdialysis, mean (SD)
Sex		
Male	0.608 (0.49)	0.608 (0.49)
Female	0.392 (0.49)	0.392 (0.49)
Age group, y		
<18	0.017 (0.13)	0.014 (0.12)
18-24	0.030 (0.17)	0.028 (0.16)
25-34	0.059 (0.24)	0.055 (0.23)
35-44	0.146 (0.35)	0.132 (0.34)
45-54	0.310 (0.46)	0.292 (0.45)
55-64	0.437 (0.50)	0.459 (0.50)
Plan type		
PPO	0.519 (0.50)	0.521 (0.50)
POS	0.350 (0.48)	0.352 (0.48)
HMO	0.110 (0.31)	0.103 (0.30)
Indemnity	0.010 (0.10)	0.011 (0.10)
EPO	0.011 (0.11)	0.013 (0.11)
Dependent	0.336 (0.47)	0.335 (0.47)
Self-funded	0.761 (0.43)	0.766 (0.42)
Pharmacy benefit	0.485 (0.50)	0.478 (0.50)
Mental health coverage	0.800 (0.42)	0.800 (0.39)
Months enrolled	30.0 (18.88)	42.5 (18.93)
Receives dialysis	0.000 (0.00)	0.763 (0.42)
Hospitalized	0.080 (0.27)	0.110 (0.31)
Total monthly spending, $	5025.39 (15 821.96)	19 653.51 (22 353.74)
Out-of-pocket spending, $	260.86 (674.14)	443.42 (1025.63)
Spending amount by category, $		
Outpatient dialysis	0.00 (0.00)	9679.59 (9941.57)
Nondialysis outpatient	908.82 (2970.48)	2921.89 (5926.37)
Inpatient	2585.59 (12 079.63)	3789.79 (14 282.83)
Physician services	1030.76 (2454.07)	1945.84 (3716.94)
Prescription medication	218.43 (654.90)	366.94 (885.20)
Prescription drug spending with coverage^b^	475.59 (1047.02)	816.36 (1371.46)
Patient-months, No.	148 704	161 069

Abbreviations: EPO, exclusive provider organization; HMO, health maintenance organization; POS, point of service; PPO, preferred provider organization.

^a^ Sample limited to patients missing none of the characteristics presented here and who were continuously enrolled for 12 months before the first dialysis treatment, the month of the first dialysis treatment, and the subsequent 12 months. Indicators for dialysis receipt and hospitalization are monthly. Spending variables are monthly and are winsorized at the 99th percentile.

^b^ Prescription drug spending with coverage was limited to patients continuously enrolled in a plan with a prescription drug benefit.

### Statistical Analysis

To assess how health care spending changes after the start of dialysis, we compared monthly spending for patients before and after their first dialysis treatment with a multivariate regression model using Stata, version 17 (StataCorp LLC). Unlike previous studies that compared patients with kidney failure with different patients who did not receive dialysis care,^4,8^ our longitudinal data allowed us to follow up patients receiving dialysis. In this way, we were able to control for unobserved, time-invariant differences in health across patients that may be associated with the onset of kidney failure, such as smoking, diet, or exercise habits.

In addition, we compared the distributions of total spending before and after the initiation of dialysis care by calculating the total amount of health care spending for each enrollee in the 12 months before the first month of outpatient dialysis care and in the first month of dialysis care combined with the following 11 months. We then assessed the distribution of total health care spending in the year before and after starting dialysis.

Similarly, we compared the distribution of total health care spending in the year after dialysis initiation for privately insured patients with the distribution for patients insured by Medicare using data from the US Renal Data System. All significance tests reported are 2-sided and at the 5% significance level unless otherwise noted.

## Results

Of the 12 392 enrollees included in the analysis, 7534 (61%; 95% CI, 60.5%-61.0%) were male and 4858 (39%) were female; 5415 (44%; 95% CI, 43.5%-44.0%) of patients were aged 55 to 64 years at baseline. Patients had been enrolled with their insurer for a mean of 30 months (95% CI, 29.9-30.1 months) Table 1 presents summary statistics for this sample segmented into observations before the enrollee’s first dialysis session and those that came afterwards. The most common insurance plan type was a preferred provider organization, and most plans are self-funded. Eighty percent of the patients had mental health coverage and 49% had a pharmacy benefit from the insurers in our data. We observed a pharmacy benefit for a beneficiary only if provided by an insurer reporting data to the Health Care Cost Institute. This means that patients for whom we did not observe prescription drug coverage may have employer-sponsored coverage from another insurer. For this reason, we restricted our analyses of the change in prescription drug spending to patients with a pharmacy benefit for the entirety of the sample. The total spending analysis should be thought of as a lower bound because increases in prescription drug spending for individuals without a pharmacy benefit reported in our data were ignored. We found no meaningful changes in enrollee or insurance plan characteristics after starting dialysis.

We further found that even before patients started dialysis, health care spending for this sample was relatively high, with a mean total monthly spending of roughly $5025 (95% CI, $4945-$5106). Nonetheless, spending increased sharply around the first outpatient dialysis session, with mean spending increasing to $19 654 (95% CI, $19 544-$19 763) per month. After patients started dialysis, the largest categories of spending were outpatient dialysis care and inpatient care.

Table 2 provides estimates of spending after the initiation of dialysis as well as unadjusted differences. The adjusted and unadjusted differences are quite similar, so we focus our discussion on the adjusted differences. The initiation of dialysis care was associated with an increase in monthly health care spending of $14 685 (95% CI, $14 413-$14 957). This represents 292% (95% CI, 287%-298%) more spending than the predialysis mean of $5025 per month. Other results show that health care spending was higher across all categories. Monthly spending was $9897 (95% CI, $9737-$10 058) per month higher for outpatient dialysis care, $1847 (95% CI, $1778-$1917) higher for nondialysis outpatient care, $926 (95% CI, $893-$959) higher for physician services, $1218 (95% CI, $1102-$1334) higher for inpatient care, and $332 (95% CI, $310-$355) higher for prescription drugs. Patient out-of-pocket spending was also higher by $170 (95% CI, $162-$178) per month.

**Table 2.** Adjusted and Unadjusted Differences in Spending^a^

Spending category	Monthly spending, mean (95% CI), $		Difference (95% CI), $	
	Before first session	After first session	Unadjusted	Adjusted
No.	148 704	161 069	NA	NA
Total	5025 (4945-5106)	19 654 (19 544-19 763)	14 628 (14 365-14 891)	14 685.19 (14 413-14 957)
Out-of-pocket	261 (257-264)	442 (438-448)	183 (175-190)	170 (162-178)
Dialysis	0 (0-0)	9680 (9631-9728)	9680 (9524-9835)	9897 (9737-10 058)
Nondialysis outpatient	909 (894-924)	2922 (2893-2951)	2013 (1942-2084)	1847 (1778-1917)
Inpatient	2586 (2524-2647)	3790 (3720-3860)	1204 (1095-1313)	1218 (1102-1334)
Physician services	1031 (1018-1043)	1946 (1930-1961)	915 (883-948)	926 (893-959)
Prescription drugs^b^	476 (468-483)	816 (806-826)	341 (319-363)	332 (310-355)

Abbreviation: NA, not applicable.

^a^ Estimates of level and adjusted and unadjusted change in spending after a patient’s first outpatient dialysis session. Standard errors clustered at the patient level.

^b^ Sample for prescription drug spending is limited to enrollees with a prescription drug benefit.

**Figure 1.**
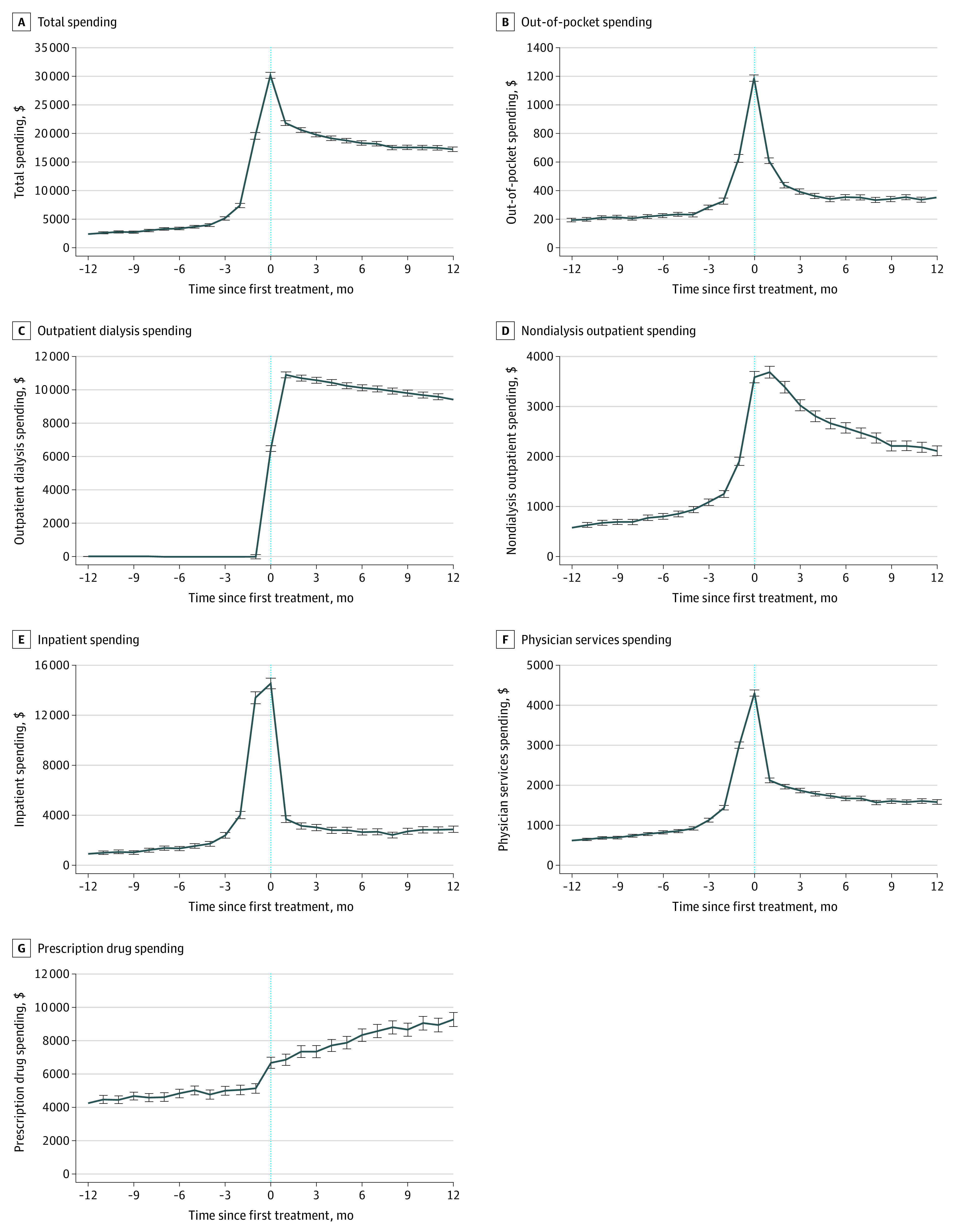
Changes in Spending at Initiation of Dialysis Care by Category Estimates of the adjusted change in spending relative to the 12 months before the patient’s first dialysis session (indicated by the vertical line) added to the mean level of spending in the reference month for total spending (A), out-of-pocket spending (B), outpatient dialysis spending (C), nondialysis spending (D), inpatient spending (E), physician services (F), and prescription drug spending limited to enrollees with a prescription drug benefit (G). Error bars give the 95% point-wise CIs. Standard errors clustered at the patient level.

Figure 1A reports the estimates of total monthly spending after the start of dialysis. We found that, although spending was slightly higher before the first month of dialysis, most of the increase occurred abruptly near the month of the first dialysis treatment. After the initial spike, spending slowly decreased to a sustained level above baseline. Our results suggest that, compared with 12 months before a patient’s first dialysis treatment, the first month of dialysis corresponded to $27 763 (95% CI, $27 239-$28 287) in additional health care spending and remained at least $15 000 (95% CI, $14 424-$15 226) higher per month throughout the next year.

Figure 1 also presents this change in different categories of spending, showing that a modest uptick in spending followed by a large and sustained increase was common across multiple types of spending (inpatient, nondialysis outpatient, and physician services). An abrupt increase in inpatient spending occurred in the month before initiating dialysis, potentially indicating that many patients in the sample may be beginning dialysis without preparatory care, with 59% (95% CI, 58%-60%) of the patients in this sample hospitalized in the same month they initiated dialysis care or the month before.

For dialysis spending, the initial increase was more stable, slowly decreasing from an increase of $10 913 (95% CI, $10 739-$11 086) to $9417 (95% CI, $9238-$9597) in the year following the first claim for dialysis. This decrease appeared to be largely noted in patients receiving transplants and ceasing dialysis care. Restricting the sample to the 92% of enrollees who did not have a claim indicating receipt of a transplant during the sample period, we found that the reduction in dialysis spending decreased from $1501 to $643. For prescription drug spending, a small increase was noted in the first month of dialysis followed by sustained increases throughout the following year to the point that the cost became $500 (95% CI, $460-$544) higher 12 months after the start of dialysis compared with the year before. Out-of-pocket spending followed a similar pattern to total, nondialysis outpatient, inpatient, and physician services spending.

**Figure 2.**
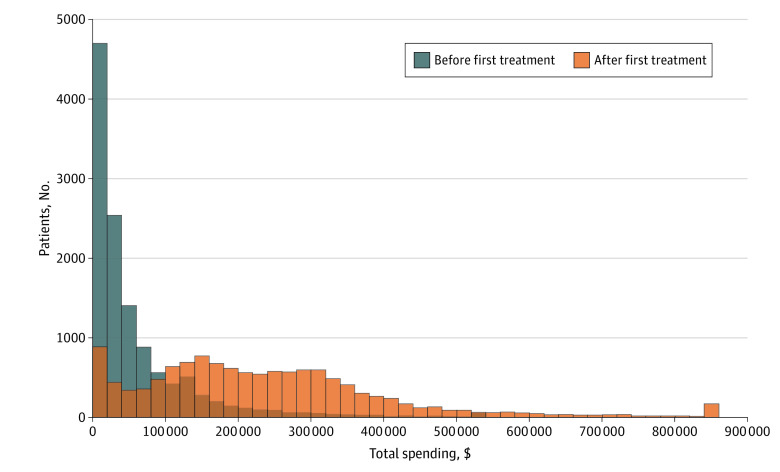
Distribution of Total Spending in the 12 Months Before and After First Dialysis Treatment Histogram bin width is $20 000. Bins are censored to lowest level of spending with fewer than 10 patients ($540 000 before treatment, $860 000 after treatment).

These estimation results report mean changes in the level of spending before and after initiating dialysis care and may obscure wide variation across patients. To examine how the entire distribution of spending changed, in Figure 2 we present the distribution of beneficiary-level total spending for both 12 months before the first dialysis claim and 12 months after (including the first month of outpatient dialysis care). We found that spending was higher in the 12 months after starting dialysis compared with 12 months before at every percentile of the distribution, and that the distribution of spending before the start of dialysis was right skewed and less than $100 000 for the most patients. The distribution after the start of dialysis, however, showed that most patients had spending between $100 000 and $300 000, with a much longer right tail of very high spending.

This difference in the distribution corresponds to very large differences in spending at the high end of the distribution, but only modest increases at the low end. For example, although the median amount of total spending increased roughly $180 029 (95% CI, $176 472-$183 586), the 90th percentile increased roughly $283 401 (95% CI, $274 420-$292 382), and the 99th percentile increased almost $494 750 (95% CI, $446 352-$543 148). These differences indicate that the initiation of dialysis care was associated not just with very large increases in spending on average, but also in exceedingly large increases for those at the top end of the distribution.

**Figure 3.**
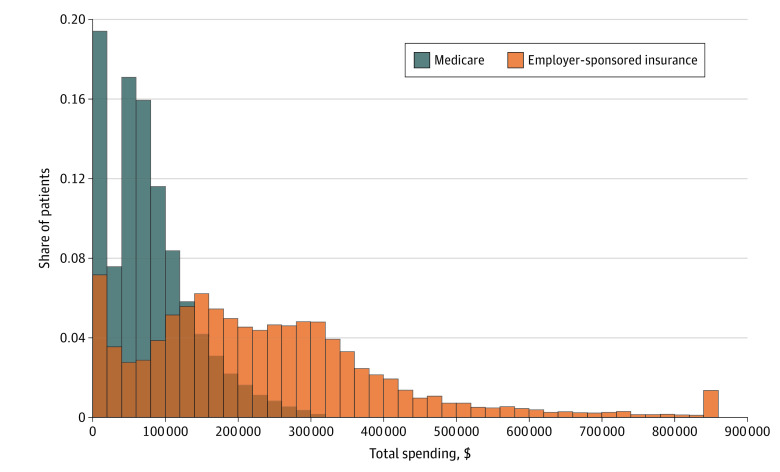
Distribution of Total Spending in the 12 Months After First Dialysis Treatment by Insurer Histogram bin width is $20 000. Bins are censored to $860 000. Sample includes Medicare enrollees in the 12 months after completing the medical evidence form and employer-sponsored insurance enrollees in the 12 months after their first dialysis treatment.

As Figure 3 shows, Medicare enrollees generally had much lower spending in the year following their first dialysis treatment than privately insured patients. The mean spending on patients with Medicare was only $80 509 (95% CI, $80 089-$80 929)—far less than the mean spending on privately insured patients of $238 126 (95% CI, $234 961-$241 291). These differences were even larger in the long right tails of the distributions: the 80th percentile of annual spending among privately insured patients was $343 082 (95% CI, $339 868-$347 275) compared with $122 384 (95% CI, $121 489-$123 080) for Medicare enrollees, the 90th percentiles were $431 666 (95% CI, $424 096-$439 919) for privately insured patients and $164 709 (95% CI, $163 614-$165 801) for Medicare enrollees and the 99th percentiles were $916 162 (95% CI, $882 260-$971 494) for privately insured patients compared with $308 314 (95% CI, $304 928-$313 168) for Medicare enrollees. As a benchmark, the 99th percentile of Medicare spending was less than the 73rd percentile for employer-sponsored insurance.

## Discussion

Previous research has shown that dialysis care imposes large costs on payers and patients. Trish et al,^7^ for example, reported that the mean monthly spending for patients with kidney failure was $13 964 higher than for other patients. Although this finding is similar to our estimated difference of $14 685 (95% CI, $14 413-$14 957), our level of spending was notably higher, at $19 654 (95% CI, $19 544-$19 763) per month after starting dialysis.

Spending is much higher for privately insured patients receiving dialysis than for those enrolled in Medicare. The US Renal Data System reports that annual per patient spending was $88 000 to $93 200 during our sample period.^5^ By contrast, the mean per-patient spending in the first year of dialysis treatment in our sample was $238 126 (95% CI, $234 961-$241 291), more than twice as high and much higher than estimates reported over a decade ago.^1^ Even examining only patients continuously enrolled in Medicare in the first 12 months after starting dialysis, when there may be additional costs from transitioning into dialysis care, we still found that spending was much higher among the privately insured cohort. The large costs borne by private insurers to cover enrollees with kidney failure underscore the importance of Medicare becoming a primary payer after 30 months.^15^

The differences in spending between enrollees receiving dialysis with private insurance and those with Medicare are especially important given growing concerns about the market power of large dialysis organizations and recent policy proposals. For instance, League et al^8^ reported that median prices per hemodialysis session for employer-sponsored plans were 6 times higher than Medicare prices, with the market power of large dialysis organizations one possible explanation for such high rates; the dialysis industry is highly concentrated, with DaVita Inc and Fresenius Medical Care combining to control a 72% share of the market.^16^ Because many insurers have limited choice for providing dialysis benefits to their enrollees, large dialysis organizations may be able to command high prices.

A recent Supreme Court ruling on the Medicare Secondary Payer Act established a path for commercial insurers to reduce their dialysis-related expenditures.^17^ The case, *Marietta Memorial Hospital Employee Health Benefit Plan v DaVita Inc*, originated from a suit brought by DaVita Inc that argued the Marietta employee health benefit plan encouraged patients to switch to Medicare by imposing high copays, coinsurance, and deductibles for dialysis, potentially violating the Medicare Secondary Payer Act. A central issue in the case was the high levels of spending for patients receiving dialysis who were enrolled in employer-sponsored health plans and whether the interpretation of the Medicare Secondary Payer Act offered by DaVita Inc would lead to a situation in which these plans no longer cover kidney failure treatments, forcing all patients with kidney failure to enroll in Medicare.^17^ This ruling potentially undermines dialysis facilities’ abilities to charge high prices and may accelerate patients’ transitions to Medicare—an outcome that could result in lower health care spending given our findings.

### Limitations

This study has limitations. First, our data do not cover all payers. Although a large share of the employer-sponsored health insurance market (the largest source of health insurance in the US) is examined in this study,^18^ our data only include claims for patients with employer-sponsored insurance offered by one of the carriers affiliated with the Health Care Cost Institute, meaning that our results may not represent the prices paid by insurers not in the Health Care Cost Institute data set. Furthermore, we could not analyze the health care spending for other insured populations, including individual market plans or Medicare Advantage, or the uninsured population. Nonetheless, to our knowledge, the data in our study represent the largest sample of private insurance claims ever used to analyze spending on patients receiving dialysis care.

The second limitation is that we cannot account for the possibility of patients’ health deteriorating for reasons unrelated to kidney failure as they initiate dialysis. Although baseline levels of health spending for patients who eventually initiate dialysis were available, we could not identify changes in health care spending caused by a patient’s health deteriorating for reasons other than kidney failure. Thus, our results may not represent the costs of initiating dialysis and holding all other health conditions constant. Nonetheless, our analysis accounts for stable, underlying health conditions of patients initiating dialysis care.

The third limitation is that we only included patients continuously enrolled in employer-sponsored insurance for 12 months before and after initiating dialysis care. Because patients receiving dialysis often lose private coverage, either due to losing their job or electing to switch to Medicare after a short waiting period, attrition from our sample was high, with 50% of enrollees with 12 months of continuous enrollment before initiation of dialysis care attritting by the end of our sample period. Because patients must maintain their employment and have the financial means to pay both the Medicare and private insurance premiums to retain employer-sponsored insurance, those who remained in our sample were likely to be healthier than those who attrit.^19^ If the patients who transition to Medicare more quickly are particularly costly to insure, our results would understate the increase in spending associated with starting dialysis and the difference between private insurers and Medicare.^15^

## Conclusions

In this cohort study, we found evidence that private insurers experienced significant, sustained increases in spending when their enrollees initiated dialysis. Dialysis care initiation was associated with an increase in total monthly spending of $14 685 (95% CI, $14 413-$14 957). This increase occurred across all categories of spending (dialysis, nondialysis outpatient, inpatient, physician services, and prescription drug) and resulted in spending much higher than was seen for patients insured by Medicare. Proposed policies aiming to limit the amount dialysis facilities charge private insurers or shift more patients receiving dialysis from private insurance to Medicare may have the potential to reduce health care spending on this high-cost population.

## Notes

Supplement. **eMethods.** Detailed Methods **eTable.** Summary Statistics Including Patients With Missing Characteristics
eReferences
