# Supplementary material for: Assessment of Spending for Patients Initiating Dialysis Care
Source: JAMA Netw Open. 2022 Oct 28;5(10):e2239131. doi: 10.1001/jamanetworkopen.2022.39131 (PMC9617169; doi:10.1001/jamanetworkopen.2022.39131)
Supplement: Supplement. — eMethods. Detailed Methods eTable. Summary Statistics Including Patients With Missing Characteristics eReferences [file jamanetwopen-e2239131-s001.pdf]

Supplementary Online Content

League RJ, Eliason P, McDevitt RC, Roberts JW, Wong H. Assessment of spending for patients initiating dialysis care. *JAMA Netw Open*. 2022;5(10):e2239131. doi:10.1001/jamanetworkopen.2022.39131

eMethods. Detailed Methods

eTable. Summary Statistics Including Patients With Missing Characteristics

eReferences

This supplementary material has been provided by the authors to give readers additional information about their work.

© 2022 League RJ et al. *JAMA Network Open.*

eMethods. Detailed Methods

Formally, we estimate the following multivariate regression model using Stata 17:

$$Y_{it} = \beta Post_{it} + \Gamma X_{it} + \gamma_i + \varepsilon_{it}, \qquad (1)$$

where Y_{it} is the level of spending on enrollee i in the month relative to his or her first treatment, t; $Post_{it}$ is an indicator variable for t being greater than or equal to 0; X_{it} is a vector of enrollee and plan controls; γ_i is a vector of enrollee fixed effects; and ε_{it} is an error term. Patient and plan controls include sex, age category, plan type, whether the plan is self-funded or fully insured, and whether the beneficiary is a dependent or the plan subscriber.

To estimate the dynamic changes in spending surrounding the initiation of dialysis care, we also estimate

$$Y_{it} = \sum_{t=-11}^{12} \beta_t v_t + \Gamma X_{it} + \gamma_i + \varepsilon_{it}, \qquad (2)$$

where v_t is an indicator variable for being t months from that patient's first dialysis treatment. In the figures that report these results, we report these estimates relative to the average level of spending 12 months prior to the initiation of dialysis care.

To compare private spending on dialysis care to patients covered by Medicare, we also use data supplied by the United States Renal Data Service.[1] This sample includes all patients continuously enrolled in Medicare for the month in which the patient completes the medical evidence form and the subsequent 11 months from 2015-2017. The medical evidence form is necessary for enrollment in Medicare due to ESRD and is typically completed at the first dialysis treatment.[2] This sample includes 2,464,675 patient months.

© 2022 League RJ et al. *JAMA Network Open.*

eTable: Summary Statistics including Patients with Missing Characteristics

	Pre-Dialysis	Post-Dialysis
	Mean (SD)	Mean (SD)
Male	0.607 (0.49)	0.607 (0.49)
Female	0.393 (0.49)	0.393 (0.49)
Age		
under 18	0.017 (0.13)	0.014 (0.12)
18-24	0.030 (0.17)	0.027 (0.16)
25-34	0.059 (0.24)	0.055 (0.23)
35-44	0.146 (0.35)	0.132 (0.34)
45-54	0.310 (0.46)	0.291 (0.45)
55-64	0.437 (0.50)	0.457 (0.50)
Plan Type		
PPO	0.517 (0.50)	0.518 (0.50)
POS	0.351 (0.48)	0.352 (0.48)
HMO	0.109 (0.31)	0.103 (0.30)
Indemnity	0.010 (0.10)	0.010 (0.10)
EPO	0.011 (0.11)	0.013 (0.11)
Dependent	0.333 (0.47)	0.332 (0.47)
Self-Funded	0.761 (0.43)	0.765 (0.42)
Pharmacy Benefit	0.486 (0.50)	0.478 (0.50)
Mental Health Coverage	0.800 (0.42)	0.800 (0.39)
Months Enrolled	29.9 (18.80)	42.4 (18.86)
Receives Dialysis	0.000 (0.00)	0.765 (0.42)
Hospitalized	0.080 (0.27)	0.110 (0.31)
Total Monthly Spending ($)	5,085.76 (15,882.56)	19,670.69 (22,366.05)
Out-of-Pocket Spending ($)	262.09 (676.48)	445.67 (1,030.57)
Spending Amount by Category ($)		
Outpatient Dialysis	0.00 (0.00)	9,674.69 (9,916.69)
Non-Dialysis Outpatient	912.79 (2,982.81)	2,925.22 (5,928.62)
Inpatient	2,605.50 (12,131.19)	3,811.59 (14,330.45)
Physician Services	1,036.68 (2,458.86)	1,950.55 (3,175.07)
Prescription Medication	219.36 (658.57)	366.12 (885.55)
RX Spending with Coverage	475.40 (1,046.42)	816.34 (1,371.41)
Patient-Months	157,496	165,204

Table note: Sample limited to patients continuously enrolled for 12 months prior to first dialysis treatment, month of first dialysis treatment, and subsequent 12 months. Sample includes patients with missing characteristics. Indicators for dialysis receipt and hospitalization are monthly. Spending variables are monthly and are winsorized at the 99th percentile. Prescription drug spending with coverage is limited to patients continuously enrolled in a plan with a prescription drug benefit.

© 2022 League RJ et al. *JAMA Network Open.*

eReferences

[1] U.S. Renal Data System. 2020 USRDS annual data report: Epidemiology of kidney disease in the United States. National Institutes of Health, National Institute of Diabetes and Digestive and Kidney Diseases, Bethesda, MD, 2020.

[2] Villani, V, Bertuzzi, L, Butler, G, Eliason, P, Roberts, JW, DePasquale, N, Park, C, McElroy, LM., McDevitt, RC. Provision of Transplant Education for Patients Starting Dialysis: Disparities Persist. Working paper. 2021.

© 2022 League RJ et al. *JAMA Network Open.*
